# Tensile Properties and Corrosion Behavior of Extruded Low-Alloyed Mg–1Sn–1Al–1Zn Alloy: The Influence of Microstructural Characteristics

**DOI:** 10.3390/ma11071157

**Published:** 2018-07-06

**Authors:** Weili Cheng, Yao Zhang, Shichao Ma, Srinivasan Arthanari, Zeqin Cui, Hong-xia Wang, Lifei Wang

**Affiliations:** 1 Shanxi Key Laboratory of Advanced Magnesium-Based Materials, Taiyuan University of Technology, Taiyuan 030024, China; cuizeqin@tyut.edu.cn (Z.C.); wanghxia1217@163.com (H.-x.W.); 2 School of Materials Science and Engineering, Taiyuan University of Technology, Taiyuan 030024, China; zhangyao391@yeah.net (Y.Z.); mashichao129@126.com (S.M.); 3 School of Materials Science and Engineering, Seoul National University, Seoul 08826, Korea; asrini@snu.ac.kr

**Keywords:** magnesium alloy, extrusion, texture, tensile property, corrosion behavior

## Abstract

A low-alloyed Mg–Sn–Al–Zn system was developed and successfully fabricated through the extrusion process. The dependence of tensile properties and corrosion behavior on microstructural characteristics of the studied alloy has been investigated. After extrusion, the alloy consists of fine dynamically recrystallized (DRXed) grains of ~2.65 μm and strongly textured coarse unDRXed grains. As a consequence, the extruded alloy showed a high-tensile yield strength (YS) of 259 MPa, ultimate tensile strength (UTS) of 297 MPa and elongation (EL) of 19.0%. The strengthening response was discussed in terms of grain size, texture and solutes. The as-extruded alloy presented severe pitting corrosion and the dependence of corrosion properties on the crystallographic orientation and the formation of corrosion products was analyzed.

## 1. Introduction

Mg alloys exhibit insufficient corrosion resistance in the body fluid due to active electrochemical potential. Alloying has been widely considered as one of potential means of improving corrosion resistance of Mg alloys. Recently, Sn has become a significant alloying element for Mg alloys on account of its similar achieved effect in term of strengthening and heat -resistance compared with rare earth (RE) elements, thus Sn-containing Mg alloys with high performance have broad engineering prospects [1,2]. The corrosion behavior of the Sn-containing Mg alloys have been reported and one of the notable points was that solutionised Sn had an effect to influence the cathodic reaction (H_2_ evolution rate) [3]. On the other hand, previous reports indicated that the second phases act as cathodic phase, which accelerates the corrosion dissolution of Mg substrate [4]. Similarly, Zeng et al. [5] have reported that the decrease in fraction of the second phase could bring about an enhancement in corrosion resistance of Mg alloys. Therefore, more care needs to be taken to avoid galvanic corrosion between the precipitate and α-Mg matrix.

Dilution of the alloying elements could be an effective consideration to promote the corrosion resistance and extrudability of Mg alloys due to the elimination of the secondary phases with low melting point [6]. Furthermore, Jiang et al. reported that the low-alloyed Mg–Zn–Ca–Mn alloy exhibited a tensile yield strength of 307 MPa and an elongation of 20.6% by the reason of a bimodal microstructure (∼2.3 μm) [7]. In addition, the deformation texture in the Mg–0.4Zn–0.1Ca and Mg–0.4Zn alloys under similar cold rolling conditions were discussed [8]. The results indicated that the different texture evolution was caused by variation in twinning behaviors, dislocation slip. So far, the effect of microstructural characteristics on the corrosion behavior of low-alloyed Mg alloys has not been clearly understood. Therefore, a low-alloyed Mg–Sn–Al–Zn alloy was subjected to extrusion to achieve a textured structure with DRXed and unDRXed grains. The resultant tensile and corrosion properties were discussed in terms of grain size, texture, solutes and/or corrosion products.

## 2. Experimental Procedure

### 2.1. Specimen Preparation

An ingot with an actual composition of Mg-0.92 wt % Sn-0.86 wt % Al-0.83 wt % Zn was prepared. Details of the raw material casting procedure have been depicted elsewhere [9]. After casting, the dimensions of the samples were 40 mm in diameter and 50 mm in length. Homogenization was carried out at 350 °C for 3 h and then 500 °C for 5 h with water quenching. An extrusion experiment was implemented at an initial casting temperature of 300 °C, a ram speed of 0.1 mm s^−1^, and an extrusion ratio of 16.

### 2.2. Microstructural Characterization

The microstructural characteristics of the samples were observed using an optical microscope (OM, Leica DM-2700M, GmbH, Wetzlar, Germany), and a scanning electron microscope (SEM, TESCAN MIRA, Brno-Kohoutovice, Czech Republic) equipped with an energy dispersive spectrometer (EDS). The alloy phases were determined by X-ray diffraction (XRD, Cu-Kα, Y-2000, Dandong Ray Instrument Co., Ltd., Dandong, China). EBSD (Electron Backscattered Diffraction) analysis was carried out using HKL EBSD detector) Oxford Instruments, Oxford, England）and HKL Channel 5 acquisition software on a field-emission scanning electron microscope (Carl Zeiss CrossBeam 1540EsB, Jena, Germany). The average grain sizes and the chemical composition of the surfaces of the studied alloys were examined several optical micrographs using the software Nano Measurer 1.2 and using an X-ray photo-electron spectroscopy (XPS, K-Alpha). Details of the test procedure have been depicted elsewhere [9]. The data were analyzed with Avantage software (5.52, Thermo Fisher Scientific, Waltham, MA, USA).

### 2.3. Tensile Properties

Tensile specimens with dimension (18 mm × 4 mm × 2 mm) were cut from the extruded rods along extrusion direction (ED). Tensile tests along the ED were then performed at room temperature using a DNS100 (SFMIT Ltd. Changzhou, China) electric testing machine with an initial strain rate of 1 × 10^−3^ s^−1^. Tensile tests were performed for three times, and the average value of these measurements was used in this study.

### 2.4. Electrochemical Test

All corrosion specimens were mechanically ground with 800–2000 grit SiC papers and polished, washed and dried completely. The specimens were encapsulated except for one end surface by an epoxy resin. The electrochemical test was conducted in simulated body fluid (SBF) solution at 37 °C. Details of the test procedure have been depicted elsewhere [9].

## 3. Results and Discussion

### 3.1. Microstructural Characteristics Prior to Corrosion

**Figure 1:**
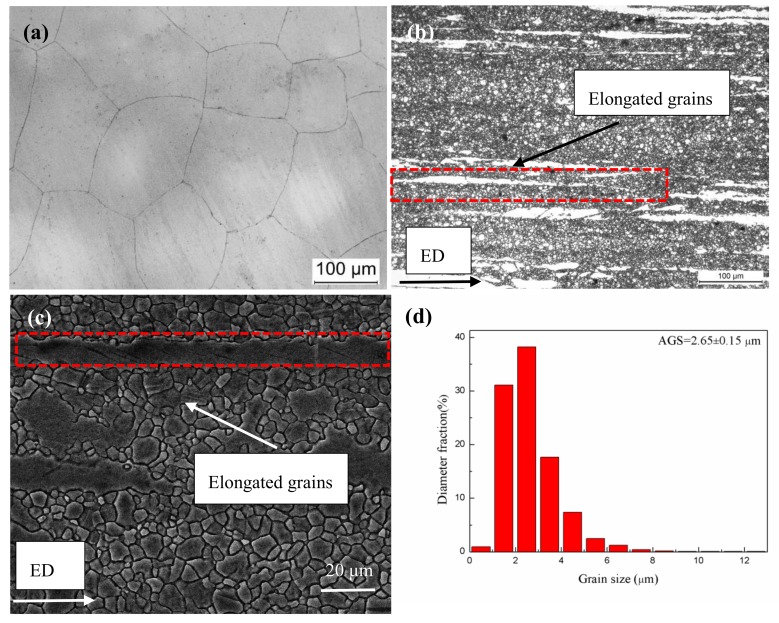
(**a**) Optical microscope (OM) micrograph of homogenized alloy, (**b**–**d**) OM and scanning electron microscope (SEM) micrographs, dynamic recrystallized (DRXed) grain-size distribution map of extruded alloy.

Figure 1 shows the micrographs of the homogenized and extruded alloys. The homogenized alloy (Figure 1a) exhibited a coarse-grained structure, with average grain size being 121.07 ± 32.15 μm. After extrusion, the structure of the alloy was greatly refined due to the dynamic recrystallization (DRX) process during the extrusion, with the average DRXed grain size of 2.65 ± 0.15 μm. Some elongated grains (unDRXed) along ED, as indicated by red dotted rectangles, could also be observed (Figure 1b,c), the fraction of DRXed grain (F_DRX_) could be determined to be 88.2% (Figure 1d). Generally, dislocation density has a great effect on the DRX mechanism. Discontinuous dynamic recrystallization (DDRX) and continuous dynamic recrystallization (CDRX) are the dominant DRX modes in the wrought Mg alloys. Our previous report indicated that the DRX mechanism in Mg–Sn–Al–Zn alloy system extruded in the same condition is dominated by continuous dynamic recrystallization (CDRX) [10].

### 3.2. Phase Composition

**Figure 2:**
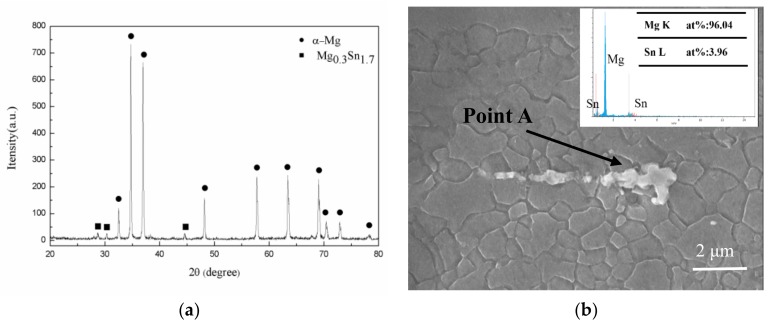
(**a**) X-ray diffraction pattern and (**b**) secondary phase with energy dispersive spectrometer (EDS) result of the extruded alloy.

The phase composition of the extruded alloy was characterized by X-ray diffraction and the corresponding result is presented in Figure 2. Note that only the diffraction peaks of α-Mg and Mg_0.3_Sn_1.7_ were observed, and no Zn or Al-containing phase occurred. Based on the SEM and EDS result of point A and XRD result, the strip-like phase could be indexed as Mg_0.3_Sn_1.7_ (Point A), which was possibly undissolved particles prior to extrusion. These particles were broken into fragments and aligned along ED, and a similar phenomenon was reported in many previous reports [1,11].

### 3.3. Texture

**Figure 3:**
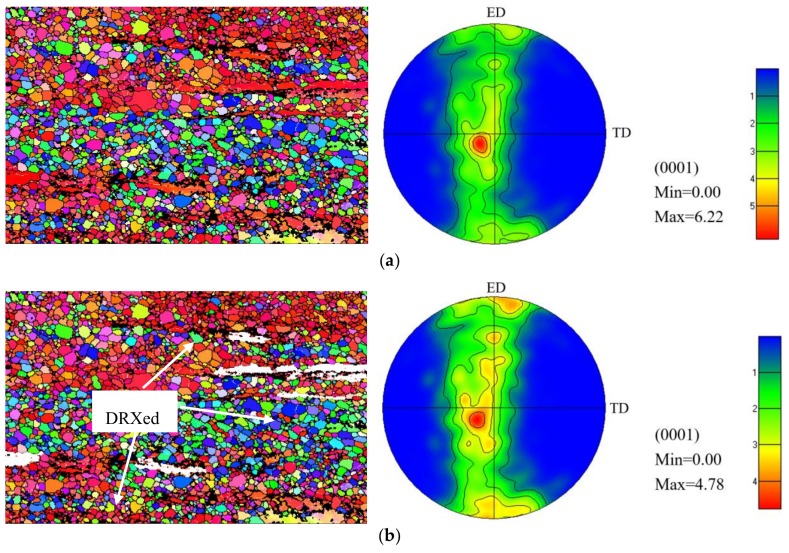

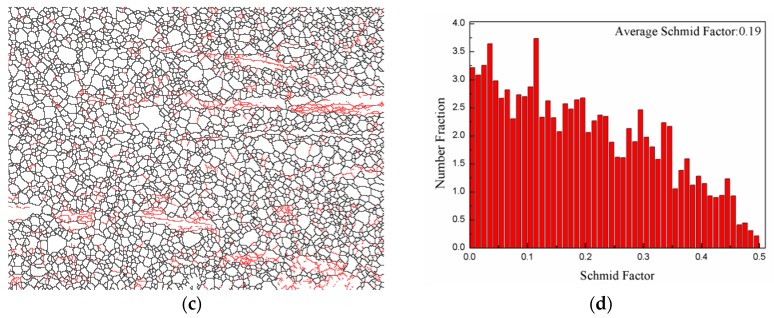
(**a**) EBSD (Electron Backscattered Diffraction) orientation maps and pole figures of the extruded alloy with and (**b**) without elongated grains, (**c**) misorientation angle distribution, (**d**) Schmid factor distribution map.

Figure 3 shows EBSD orientation maps and (0001) pole figures of the extruded alloy. As indicated, a type of basal texture in which basal planes are preferentially parallel to the ED can be determined. In addition, the grains composed of both recrystallized and elongated grains (Figure 3a,b) have a stronger texture than the grains consisting of only recrystallized grains, suggesting that the elongated grains retain strong texture. Figure 3d shows the misorinentation angle distribution of the alloy, which consists of low angle grain boundaries (red line, with misorientation of <5°) and high angle grain boundaries (black line). The average Schmid factor (Figure 3d) for the basal slips of the extruded alloy is 0.19, indicating that the unDRX grains contribute strongly to the tensile yield strength along the ED. Similar results were also reported in [11].

### 3.4. Tensile Properties

**Figure 4:**
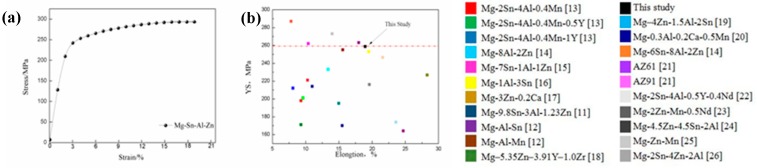
(**a**) Engineering stress-strain curve and (**b**) yield strength (YS) and elongation (EL) of various Mg-based extruded alloys.

The tensile stress-strain curve of the as-extruded alloy is presented in Figure 4a. The result shows that yield strength (YS), ultimate tensile strength (UTS) and elongation (EL) of the as-extruded alloy are 259 MPa, 297 MPa and 19.0%, respectively. The reported EL and YS of the studied alloy and other extruded Mg alloys are shown in Figure 4b. It could be inferred that the combined tensile properties of the studied alloy were superior than other extruded Mg–Al and/or Mg–Zn based alloys studied in References [11,12,13,14,15,16,17,18,19,20,21,22,23,24,25,26]. For instance, the YS of the extruded alloy is 47, 58, 41 and 32.3, 64 MPa higher than that of Mg–Al–Sn based alloys and Mg–Zn in [11,16,18,23]. Even some reported Mg–Al based alloys present similar YS with the present extruded alloy, while the former ones exhibit poor elongation [11,15].

The YS of the Mg alloys was determined by the grain size, texture and solute atoms. Thus, for the extruded alloy, the improvement of YS is mainly on account of grain boundary strengthening (*σ_gb_*), solid solution strengthening (*σ_ss_*) and texture strengthening (*σ_tex_*).

The YS could be expressed as:

(1) $$\sigma_{0.2}=\sigma_{gb}+\sigma_{ss}+\sigma_{tex}$$

the effect of the grain size on YS can be calculated using Hall–Petch equation [27]:

(2) σgb=σ0+kd−12

where *σ_gb_* is the increase yield stress by reason of grain boundary strengthening, *σ*_0_ is the material constant (*σ*_0_ = 21 MPa) [17], *k* is the Hall–Petch coefficient (*k* = 280 MPa(µm^−½^)) for the Mg–Sn based alloy; the average grain size is 2.65 µm, so the *σ_gb_* is approximately 193.0 MPa.

Almost all the alloy elements are homogenized into the Mg matrix, and according to solid solution strengthening model of materials proposed by Gypen and Deruyttere [28], the *σ*_ss_ can be describe as:

(3) σss=(kSn1nCSn+kAl1nCAl+kZn1nCZn)n

where *n* is a constant with 2/3, *k_Sn_*, *k_Al_* and *k_Zn_* are the strengthening constants for solutes Sn, Zn and Al, respectively [29]. The *C_Sn_*, *C_Al_* and *C_Zn_* are the concentration of solutes Sn, Al and Zn in at %, with values being 2.05 × 10^−3^, 9.03 × 10^−3^, 3.75 × 10^−3^ respectively. Consequently, the contribution of YS from solid solution strengthening is about 27.3 MPa.

The influence of texture to YS (*σ_tex_*) can be expressed as:

(4) $$\sigma_{tex}=M_{\tau 0}$$

where *M* is an orientation factor for the basal texture in the alloy, and *τ*_0_ is the critical resolved shear stress (CRSS) related to the operative slip system. Previous research suggested that, for Mg–Sn based alloys, the value of *M* could be calculated by taking 6.5 times the maximum texture intensity (6.22) [29]. Based on the previous work, the value of *τ*_0_ is ranged from 0.45 to 0.87 for basal slip in pure Mg [30]; in this work, because of the low content of alloy elements, it was supposed to be 0.66 based on the information of pure Mg. As a result, the relative contributions from texture strengthening is about 26.7 MPa.

In sum, the grain boundary is the dominant strengthening mechanism and the solution as well as texture strengthening could not be negligible. Due to the deviations of the variables used in the equations and possible shortcomings in prepared materials, the calculated value could not meet the experimenalt value.

### 3.5. Corrosion Tests in the Simulated Body Fluid (SBF) Solution

**Figure 5:**
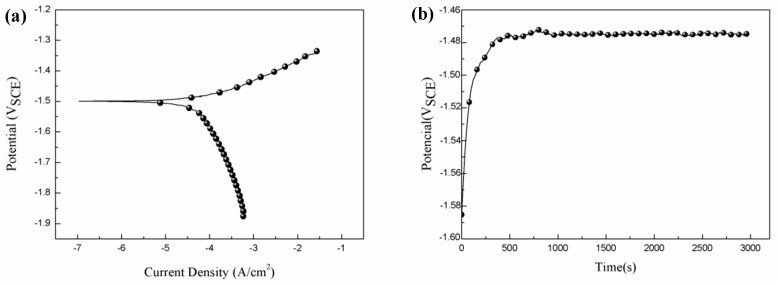
(**a**) The potentiodynamic polarization curve and (**b**) the open circuit potential of the extruded alloy measured in the simulated body fluid (SBF) solution.

Figure 5a shows the potentiodynamic polarization curve of the extruded alloy. It exhibits a dissymmetrical shape, from which it can be found that the increased rate of current density in the anodic branch is much faster than that of the cathodic branch. In a general way, the cathodic polarization curve is assumed to indicate hydrogen evolution via solution reduction, and the anodic polarization curve exhibits the dissolution of Mg substrates [4].

The fitted corrosion parameters and calculated corrosion rate of as-extruded and as-cast Mg–1Sn–1Al–1Zn alloys are listed in Table 1, where *β_a_* and *β_c_* are the anodic and cathodic Tafel slope, respectively, and *i_corr_* (the current density) is related to the average corrosion rate (*P_i_*, mm/a) according to the following equation [31]:

(5) $$P_{i}=22.85i_{corr}$$

As shown in Table 1, the average corrosion rate of as-extruded alloy is relativly low compared to that of as-cast Mg–1Sn–1Al–1Zn alloy.

Figure 5b shows the OCP (open circuit potential) curve immersed in SBF solution. The OCP value of the alloy was primitively about −1.59 V_sce_, and then increased quickly and undulated between −1.47 V_sce_ and −1.49 V_sce_, which reveals that the alloy was in a steady state after 1000 s of immersion. Previous research suggested that the higher steady OCP value was owing to the formation of more compact surface layer [3].

**Figure 6:**
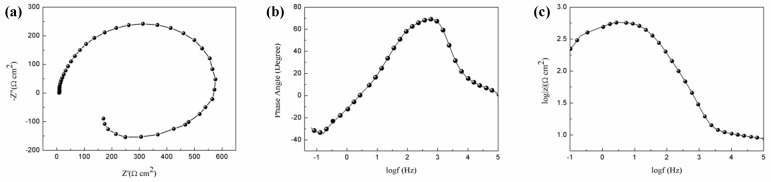
(**a**) Nyquist plot, (**b**) Bode plot of impedance vs. frequency, (**c**) Bode plot of phase angle vs. frequency of the extruded alloy.

Figure 6 shows the Nyquist plot, Bode plot of impedance vs. frequency, as well as frequency vs. degree. As exhibited, the studied alloy consisted of a capacitive loop at the high frequency and an inductance loop at the low frequency. The capacitive loop at the high frequency zone indicates the charge transfer reaction at the sample surface and electrode, and the dimension of capacitance loop determines the charge transfer resistance. In addition, the low-frequency inductance loop can also be observed, implying that the surface film is not compact and broken down [5]. It should be mentioned that the value of the impedance modulus (e^2.7^) of the studied alloy is approximately one order of magnitude lower than that of the previous reported Mg–2Sn–6Bi alloy (e^3.5^) [9], which may be ascribed to the difference in solutionised elements, texture as well as the corrosion products.

**Figure 7:**
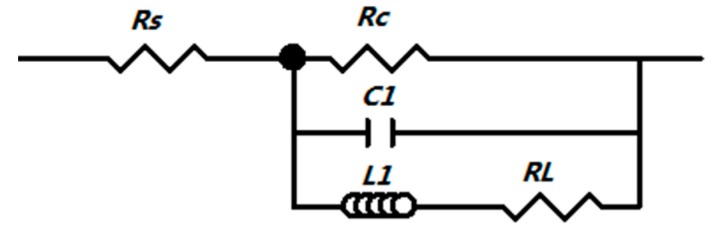
Equivalent circuit of the EIS (Electrochemical impedance spectrum) spectra for as-extruded and as-cast alloys.

In order to further expound the corrosion character of the as-cast/extruded Mg–1Sn–1Al–1Zn alloy, electrochemical equivalent circuit model is built to simulate the Nyquist plot and the Bode plot in Figure 7. There are two parts in the equivalent circuit. The first part (*R*1) is resistance of solution. The second part (*R*2) is a charge transfer resistance in parallel with *C*1, which represents the constant phase element, and an inductance *L*1 in series with an inductive resistance *R*3, which is the resistance of membrane. The fitted parameters are shown in Table 2. The large resistance value indicates the lower dissolution rate of Mg matrix and the lower *C* value indicates a more protective surface [5]. In general, the polarization resistance, *R_p_*, can be determined by the following equation [9]:

(6) $$R_{p}=R_{s}+R_{c}+R_{L}$$

where *R_s_* represents the solution resistance, *R_c_* is the change transfer resistance of the corrosion process on the surface, and *R_L_* represents inductance resistance. As shown in Table 2, the *R_p_* value of the extruded alloy is much higher than that of as-cast Mg–1Sn–1Al–1Zn alloy.

**Figure 8:**
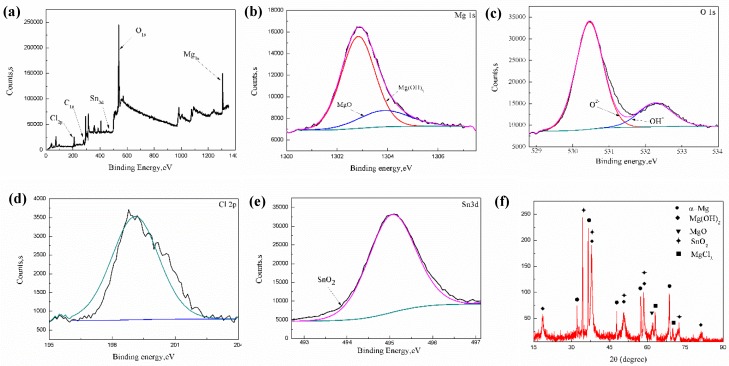
X-ray photo-electron spectroscopy (XPS) and X-ray diffraction (XRD) analysis formed oxide film on the extruded alloy surface: (**a**) survey scanning spectrum, (**b**) high-resolution Mg_1s_ spectrum, (**c**) high-resolution O_1s_ spectrum, (**d**) high-resolution Cl_2p_ spectrum, (**e**) high-resolution Sn_3d_ spectrum. (**f**) XRD pattern of corrosion products.

The XPS and XRD analyses of the extruded alloy immersed in SBF solution for 40 min are shown in Figure 8, which reveal the presence of Mg, O, Cl and Sn elements. As indicated, the Mg_1s_ spectrum can be segmented into two peaks. The binding energy at 1304 eV is attributed to MgO, with the binding energy at 1303 eV corresponding to Mg(OH)_2_ [32]. It can be seen that the O_1s_ spectrum constituted two peaks, the oxygen in the oxide at 530.6 eV matches MgO and SnO_2_ [9], and the oxygen at 532.6 eV refers to Mg(OH)_2_ [33]. Moreover, as shown in Figure 8d, the binding energy of Cl_2p_ was about at 199 eV in connection with the formation of MgCl_2_ in the MgO/Mg(OH)_2_ layered structure by the reaction with Cl_2p_. In Figure 8e, the binding energy of Sn_3d_ at 495 eV was SnO_2_.

Because Mg alloys are sensitive in corrosion media, the electrochemical corrosion reaction can take place effortlessly in SBF solution.

(7) $$\mathrm{Anode}\;\mathrm{regions}:\mathrm{Mg}+H_{2}O\to Mg^{2+}+OH^{-}+\frac{1}{2}H_{2}+e^{-}$$

(8) $$\mathrm{Cathode}\;\mathrm{regions}:H_{2}O+e^{-}\to OH^{-}+\frac{1}{2}H_{2},Mg^{2+}+2OH^{-}\to Mg{\left(OH\right)}_{2}$$

It was reported that Sn can react to produce SnH_4_ in the cathode region [34]. SnH_4_ can react with water forming SnO_2_ through the following equation:

(9) $$\mathrm{Sn}+4H_{2}O\to \mathrm{Sn}H_{4}+4OH^{-}$$

(10) $$\mathrm{Sn}H_{4}+2H_{2}O\to \mathrm{Sn}O_{2}+3H_{2}$$

The Mg^2+^ could combine with OH^−^ to form insoluble Mg(OH)_2_ and deposit on the surface of the alloy. The dissolution of corrosion products and preferential formation of the more soluble magnesium chloride could occur via the following reaction:

(11) $$Mg^{2+}+2Cl^{-}\to \mathrm{MgC}l_{2}$$

Thus, it can be summarized that the corrosion product film consisted of MgO, Mg(OH)_2_, SnO_2_ and the resultant reaction product of MgCl_2_, XRD results, as shown in Figure 8f, also validated the formation of the aforementioned corrosion products.

**Figure 9:**
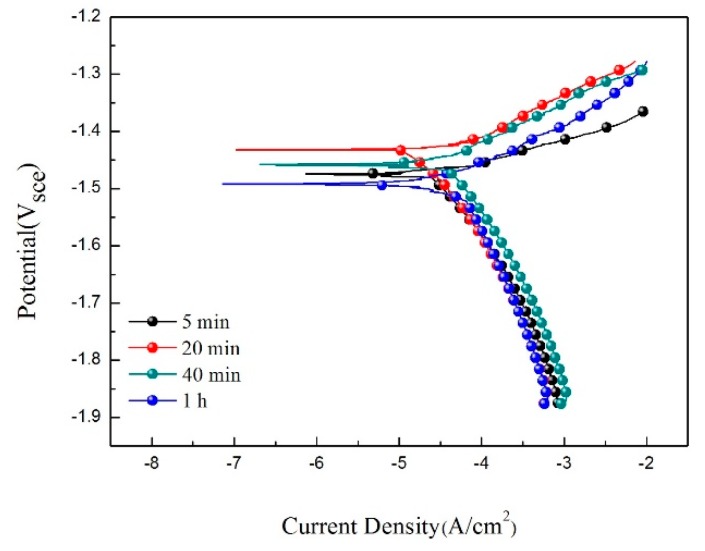
Potentiodynamic polarization curves of samples immersed in SBF solution for different time intervals.

Figure 9 shows the potentiodynamic polarization curves of the extruded alloy after immersion in SBF solution at different times, and the fitting results are listed in Table 3. It can be seen that the *E_corr_* potential increased first and then decreased as the immersion time increased, and the *i_corr_* achieved the lowest point at 20 min. The main reason for the decreasing *i_corr_* value is the protective corrosion product film which formed in the reaction [30]. As the reaction time increases, the corrosion product film was broken down while the *i_corr_* value was raised.

**Figure 10:**
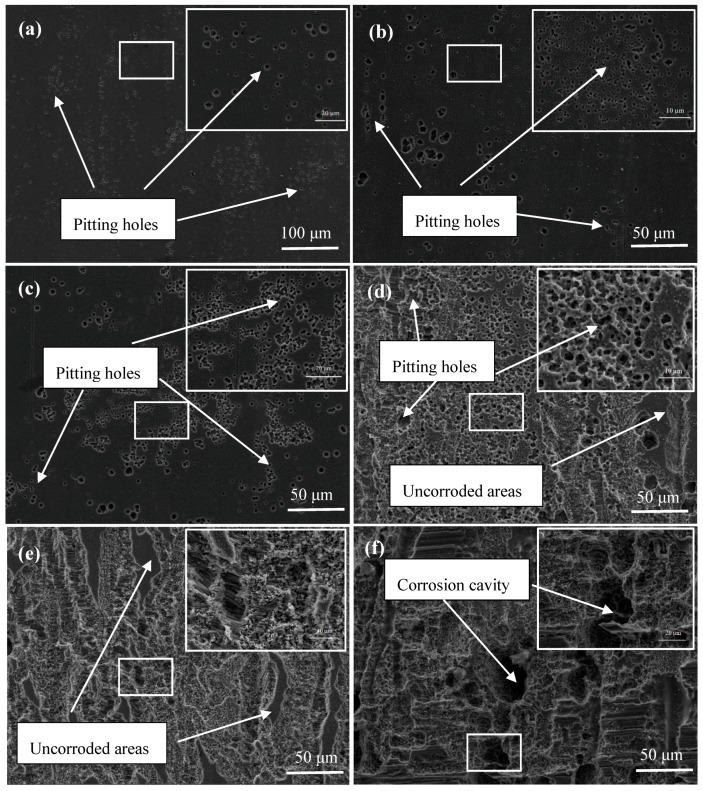
SEM micrographs showing the surfaces of the alloy removing corrosion products after being immersed for (**a**) 5 min, (**b**) 10 min, (**c**) 20 min, (**d**) 40 min, (**e**) 1 h and (**f**) 3 h after immersion in the SBF solution.

In order to inquire into the corrosion process, the SEM micrographs of the extruded alloy surfaces in the SBF solution at 37 °C for different times (after removal of corrosion products) are presented in Figure 10. As exhibited, the localized corrosion with small pitting was observed after 5 min immersion. After 20 min immersion, the small pitting holes became joined together and more tiny holes could be observed in the surface. Between 20 and 40 min, the corrosion became more intense and obvious, and almost all the observed surfaces were corroded. This phenomenon agreed well with the experimental results in Table 2. Note that the pitting holes were preferentially distributed along partial DRX and/or elongated grain boundaries (Figure 10a,b), which possibly related to the grains including low-angle grain boundaries as shown in Figure 3c. Similarly, previous report validated that the presence of grain boundaries or subgrain boundaries will be preferentially attacked at the initial stage [35]. Furthermore, with the increased reaction time, the corrosion cavities could be observed on account of the formation of cracks by making the solution reach the matrix [13]. Finally, the deeper corrosion cavities were formed as shown in Figure 10f. It should be noticed that some uncorroded regions which are similar to the original matrix morphology without any pitting hole or crack could also be observed. It is interesting to find that the uncorroded regions correspond to the elongated grain regions as shown in Figure 1b,c.

As mentioned above, the base plane of grains with red color is parallel to the ED, namely, basal grains (Figure 3a). Many previous reports validated that the corrosion rates increased in the order (10$\overset{-}{1}$2) < (11$\overset{-}{2}$3) < (10$\overset{-}{1}$0) < (11$\overset{-}{2}$0) < (0001) in Mg and Mg alloys. Such orientation-dependence may emanate from the variation in surface energy and surface atomic-packing density [36]. Furthermore, Hagihara et al. and Jang et al. indicated that the variation in plane atomic packing density and chemical bonding strength with surface orientation affected *Rc* via variations in the atomic bonding strength on the surface [36,37]. In addition, the surface energy for Mg (0002), (11$\overset{-}{2}$0) and (10$\overset{-}{1}$0) planes are 15.4, 30.4 and 29.9 kJ mol^−1^, respectively [38]. Thus, the (0002) planes dissolve more slowly than the (11$\overset{-}{2}$0) and (10$\overset{-}{1}$0) planes, which brings about a lower corrosion rate of Mg alloys. Recently, Song et al. [35] also found that grains with a basal orientation were more steady, and had lower corrosion rates than those with a non-basal orientation. Similarly, in this study, the formation of certain pitting holes on the surface could be ascribed to the preferential dissolution of non-basal grains. It should be deduced that some elongated grains with strong basal texture (red colored regions in Figure 3) was possibly related to the formation of elongated uncorroded areas in Figure 10d,e.

**Figure 11:**
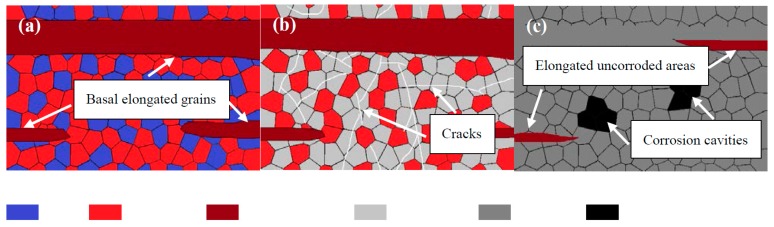
Schematic illustration of corrosion mechanism of the alloy in SBF solution (**a**) at initial time, (**b**) at middle time, (**c**) at final time.

Figure 11 is a schematic illustration of the corrosion mechanism of the extruded alloy in SBF solution. As shown in Figure 11a, when Mg substrates were exposed to the solution initially, the non-basal grains could absorb and violently react with H_2_O preferentially. The presence of Mg and Sn react with H_2_O to produce Mg(OH)_2_ and SnH_4_ in the cathode region (7, 9), and the Mg substrates immediately translated to Mg^2+^ and contacted with the solution and accompanied the hydrogen evolution reaction (8). After a prolonged immersion time, a layer of corrosion products film was formed in the corroded area (Figure 11b french gray part); at the same time, some basal grains have already begun to react with the solution, and then cracks occur on the corrosion products. The cracks led to the SBF solution contact with the fresh matrix; SnH_4_ can react with H_2_O forming SnO_2_ through Equation 10. With further immersion , these cracks could transport Cl^−^ permeating through the film structure to react with Mg(OH)_2_, causing the prior adsorption and the OH^−^ replacement by the Cl^−^ ions (11). Therefore, the fresh matrix will generate nucleation for pitting corrosion and be further corroded. Finally, almost the entire surface had been corroded [39], with black corrosion cavities (Figure 11c) on the dark gray substrate and some uncorroded areas corresponding to the basal elongated grains.

To sum up, the preferential etching of non-basal grain will accelerate the overall process of corrosion [36]. Even though there is the existence of a very low fraction of Mg_0.3_Sn_1.7_, the effect of precipitates in the α-Mg matrix on corrosion properties can be said to be negligible. In SBF solution, ions such as Cl^−^ have strong acidic anions, and films formed at the beginning cannot effectively protect the substrate. Therefore, the corrosion process will continue to occur [35]. Combined with XPS and XRD analysis, there is no product including Al and Zn formation, which shows that these elements have little effect on corrosion.

## 4. Conclusions

(1) The average grain size of homogenized alloy was 121.07 ± 32.15 μm. After extrusion, the alloy exhibited a microstructure consisting of fine dynamically recrystallized (DRXed) grains of ~2.65 μm and coarse unDRXed grains with strong texture.
(2) The extruded alloy showed a high YS of 259 MPa, UTS of 297 MPa, and EL of 19.0%, which was ascribed to the grain boundary, solid solution and texture strengthening as well as a moderate SF value.
(3) The presence of non-basal grains in the extruded alloy accelerate the corrosion process of the present extruded low-alloyed Mg–Sn–Al–Zn alloy. In addition, the corrosion product film mainly consisted of MgO, Mg(OH)_2_ and MgCl_2_, which cannot protect the matrix effectively.

## Figures and Tables

**Table 1:** Electrochemical parameters of the as-extruded and as-cast alloys obtained from the potentiodynamic polarization test.

Specimen	*E*_corr_ (V_sce_)	*i*_corr_ (mA/cm^2^)	*β_a_* (mV)	*β_c_* (mV)	*P_i_* (mm/a)
As-extruded	−1.48	4.93 × 10^−2^	54.71	382.29	11.2
As-cast	−1.53	5.42 × 10^−2^	167.3	228.63	12.4

**Table 2:** Fitting results of the EIS of the as-extruded and as-cast alloys.

Specimen	*R_S_* (Ω cm^2^)	*C*1 (F/cm^2^)		*R_C_* (Ω cm^2^)	*R_L_* (Ω cm^2^)	*L*1 (H/cm^2^)	*R_P_* (Ω cm^2^)
		*C*1	*n*				
As-extruded	9.741	6.818 × 10^−^^6^	0.995	526.5	187.7	417.1	723.94
As-cast	1.663	5.655 × 10^−5^	0.852	110.3	75.46	6.77	187.33

**Table 3:** Fitting results of the polarization curves.

Specimen	*E*_corr_ (V_sce_)	*i*_corr_ (mA/cm^2^)	*β_a_* (mV)	*β_c_* (mV)	*P_i_* (mm/a)
5 min	−1.49	4.83 × 10^−2^	66.74	270.19	11.0
20 min	−1.43	2.93 × 10^−2^	62.40	348.26	6.6
40 min	−1.46	4.12 × 10^−2^	77.62	211.71	9.4
1 h	−1.49	4.80 × 10^−2^	80.06	252.76	10.9
